# Keep Your Hopes Up: An Examination of Racial Differences in the Association Between Hope and Pain

**DOI:** 10.1093/geroni/igab046.3232

**Published:** 2021-12-17

**Authors:** Kyrsten Hill, Emily Behrens, Dylan Smith, Jason DeCaro, Brian Cox, Patricia Parmelee

**Affiliations:** 1 The University of Alabama, Tuscaloosa, Alabama, United States; 2 University of Alabama, Alabama, United States; 3 Stony Brook University, Stony Brook, New York, United States; 4 University of Alabama, Tuscaloosa, Alabama, United States; 5 University of Alabama, University of Alabama, Alabama, United States

## Abstract

Hope has been associated with increased pain tolerance (Snyder et al., 2005) and has been incorporated in interventions targeting chronic pain (Howell et al., 2015; Katsimigos et al., 2020). Research suggests that African Americans with osteoarthritis (OA) pain experience greater pain severity and disability compared to non-Hispanic White individuals (Vaughn et al., 2019). Although the literature is limited, there is some evidence to suggest racial/ethnic differences in hope (Chang & Banks, 2007). The current study examined race as a moderator of the association between hope and pain in a sample of older adults. Experience sampling (ESM) data was used from a multi-site study examining non-Hispanic White and African American individuals with knee osteoarthritis (OA). Participants completed the Adult Hope Scale (Snyder et al., 1991) during baseline interviews and self-reported momentary pain during 28 ESM calls. Multilevel models revealed a significant interaction between hope and race (p = .04). Specifically, greater hope was associated with decreased momentary pain, and this association was stronger for African American compared to non-Hispanic White individuals. Results suggest that high levels of hope may be particularly protective for African American chronic pain patients. These findings can help inform existing and future interventions focused on enhancing hope in chronic pain populations. (Supported by AG041655, P. Parmelee and D. Smith, Co-PIs)

